# 3D Pose Estimation Using Virtual Projection Based on 3D Reconstructed Model

**DOI:** 10.3390/s26113302

**Published:** 2026-05-22

**Authors:** Jung-Woo Kim, Sol Lee, Byung-Seo Park, Hak-Bum Lee, Dong-Ho Kang, Young-Ho Seo

**Affiliations:** 1 Department of Electronic Materials Engineering, Kwangwoon University, 20, Kwangwoon-ro, Nowon-gu, Seoul 01897, Republic of Korea; kjwhigherideal@gmail.com (J.-W.K.); hblee@kw.ac.kr (H.-B.L.); dhkang@kw.ac.kr (D.-H.K.); 2 Omotion Inc., 37, Maebongsan-ro, Mapo-gu, Seoul 03909, Republic of Korea; s.l@omotion.co.kr (S.L.); dev.bspark@omotion.co.kr (B.-S.P.)

**Keywords:** pose estimation, 3D skeleton, 3D mesh, 3D point cloud, volumetric capture, multi-view RGB-D, joint refinement

## Abstract

In this paper, we estimate and refine 3D human pose using the 3D point cloud or mesh model reconstructed from RGB-D cameras or volumetric capture systems. We first reconstruct the 3D model using the multi-view cameras to estimate a highly accurate skeleton. To obtain a 2D skeleton with low error, the reconstructed 3D model is projected to four virtual planes after decidi ng the direction of the 3D model. Four 2D skeletons are estimated from four images projected in the virtual plane. Afterward, the refinement process selects candidate joints based on the distribution of local vertices and the DBSCAN algorithm. It applies a sphere fitting to ensure that the final joints are located within the body volume. The joints are combined at the intersection through the back-projection of the joints, including those in the 2D skeleton on the virtual plane. The joints in the intersection are refined using the spatial distribution of the 3D information. Through the proposed method, we estimated a stable and geometrically consistent 3D human pose from reconstructed volumetric data. Using models with ground truth, we calculated the MPJPE between the skeletons of the proposed and the ground truth. The 3D pose estimation was evaluated through a visual assessment of the captured image, and the results were quantitatively compared with the 3D joint positions acquired by the motion capture device.

## 1. Introduction

Computer vision aims to realize human visual perception using a computer. Among the technologies for extracting information by analyzing images captured by a camera, it is essential to search for and estimate both the position and direction of an object. Specifically, the technique used to recognize the physical features of the human body is called human pose estimation. This estimates the position of human joints and bones in images and videos. Since not all humans in an image have enough information to find all parts of the skeleton, we do not find all skeleton information. Although a human maintains the same pose, the result of the pose estimation may differ due to the capturing viewpoint, overlapping objects, or illumination angle. The human pose estimation technique remains a challenging task, despite extensive research over a long period [1]. Until now, various studies for the pose estimation have been conducted using the rule-based method. Recently, deep learning-based studies have been actively researched and are rapidly improving performance.

We focus this study on 3D pose estimation among various pose estimation techniques. The 3D skeleton obtained through 3D pose estimation can provide more information in the 3D space than the 2D skeleton. However, in the case of using a 3D sensor, the accuracy of estimating 3D information is limited by the depth robustness of the 3D sensor. Zolfaghari et al. researched a sparse coding method to decompose samples in the 3D human pose database, allowing the dictionary to learn features that frequently appear in 3D poses [2]. Munea et al. provided a comprehensive survey of pose-estimation methods (including Bayesian/optical-flow paradigms) rather than proposing a specific new algorithm [1]. These methods have the drawback of low speed and higher errors for complicated poses. Development in deep learning has brought rapid advances in 3D pose estimation. In multi-view RGB settings, a typical pipeline first detects 2D joints and then lifts/triangulates them to 3D [3,4,5]. Instead of Unal et al. (multi-camera calibration), anatomy- or physics-aware losses have been incorporated in 3D HPE to regularize plausible poses (e.g., bone-based constraints) [6]. Most previous methods used a 2D skeleton and combined it with 3D pose information to reconstruct the 3D human pose. As an example of 2D-to-3D lifting via pairwise relations, distance-matrix regression has been explored [7]. Xu et al. proposed a geometric method for detecting joints from a single-frame point cloud captured using depth sensors [8]. Zhang et al. introduced a hierarchical context network that aggregates multi-scale features to improve pose estimation accuracy [9]. Recent studies have explored geometric and learning-based approaches for 3D human pose estimation from structured 3D data and images. Geometry-based methods utilize spatial information to estimate joint locations directly from reconstructed models, while deep learning approaches leverage contextual features for improved accuracy.

In general, multi-view methods [10,11] outperform monocular approaches [12] by reducing depth ambiguity through cross-view integration. Learnable triangulation frameworks integrate multi-view observations to reconstruct robust 3D poses [10], while recent approaches incorporate temporal reasoning to further refine pose estimation [11]. Recent state-of-the-art methods introduce high-level priors to improve performance. ActionPose incorporates action-aware constraints for motion-consistent estimation, RePOSE applies recurrent reasoning for iterative pose refinement, and PersPose introduces perspective encoding to address depth ambiguity in monocular settings [11,12,13].

Moreover, many studies based on monocular images have been conducted. Kolotouros et al. estimated the 3D pose by fitting a parametric (SMPL) body model to 2D joints with optimization-in-the-loop [14]. Wu et al. predicted explicit compositional limb depth maps from a single RGB image and lifted them to 3D [15]. Estimating 3D pose from monocular video by leveraging temporal cues is also effective [16,17]. Although such video-based methods aggregate information across frames, they remain monocular and are not equivalent to true multi-view setups.

Meanwhile, in recent studies within computer vision, hybrid approaches that combine preprocessing, domain priors, optimization, and deep learning have been actively explored. For example, Sun et al. proposed a framework that integrates heatmap-based pose estimation with optimization-based refinement to improve both accuracy and structural consistency [18]. Another study by Huang et al. incorporated geometric constraints and feature refinement strategies into deep neural networks to enhance robustness under occlusion and complex backgrounds [19]. In addition, Li et al. introduced a hybrid approach that combines temporal modeling with optimization-based correction to address nonlinear motion dynamics in human pose estimation [20]. These approaches pursue robustness and efficiency by reducing the effective search space using domain knowledge, refining the solution space through optimization, and performing learning-based inference.

We propose a geometry-driven 3D pose estimation method that operates directly on reconstructed volumetric data by combining 2D pose estimation, 3D geometric reasoning, and spatial constraint-based refinement. We extend this hybrid philosophy to the 3D model context by explicitly imposing mesh interiority and joint direction constraints through PCA-based frontal alignment, virtual plane projection, triangulation, DBSCAN, and circle or sphere fitting.

Accordingly, we propose a training-free and geometry-driven 3D skeleton extraction framework that estimates sparse joint positions directly from reconstructed point clouds or meshes, without fitting predefined parametric body models such as SMPL or SMPL-X. After determining suitable viewpoints for estimating 2D pose from the 3D point cloud generated by multiple RGB-D images, we estimate the 2D pose in each virtual viewpoint. Using the 3D model and multiple 2D skeleton distributions, we limit the region in which the 3D skeleton exists, thereby attempting to decrease the error of the 3D skeleton. The key contributions of this method are as follows.

- **Ensuring 3D consistency through direct constraints on the 3D model**: After PCA-based frontal alignment and multi-face virtual plane projection, we back-project the 2D poses from each view to form intersection regions, which yield geometrically plausible joint candidates inside the reconstructed body volume. This reduces occlusion and depth ambiguity in the evaluated setting, providing a consistent pipeline for stable 3D skeleton estimation from volumetric data.
- **Density-based refinement and modularity**: Using the bisector plane of the connected bones as a reference, we apply DBSCAN and circle or sphere fitting to remove outlier joints and refine their positions. This refinement module remains applicable even when the 2D pose detector or the joint definition changes, offering strong extensibility and reusability across different sensors and datasets.

Rather than claiming general superiority over parametric body-model fitting, this work positions the proposed method as a training-free and model-independent skeleton extraction framework that is complementary to SMPL-based approaches.

This paper is organized as follows: In Section 2, the process of obtaining a photorealistic 3D model is described, and in Section 3, a 3D pose estimation algorithm using this model is detailed. Section 4 presents the results of various experiments conducted using this algorithm. In Section 5, we review additional discussions and practical considerations related to the proposed method and its results, and Section 6 concludes this paper.

## 2. Related Works

Research on 3D human pose estimation has evolved from monocular image–based inference to multi-view triangulation and, more recently, to volumetric model–based reconstruction. This section reviews representative studies relevant to the proposed approach, focusing on 3D skeleton generation from RGB or RGB-D inputs, as well as the use of geometric or volumetric consistency constraints.

Early studies estimated 3D poses directly from 2D images by learning a mapping from 2D joint coordinates to 3D space. Zolfaghari et al. [2] introduced a sparse coding framework that decomposes 3D human pose databases into representative atoms to reconstruct poses with compact dictionaries. Munea et al. [1] provided a comprehensive survey of pose-estimation methods, summarizing classical Bayesian and optical-flow–based approaches that often suffered from slow inference and pose-dependent errors. Mei et al. [21] improved the 3D estimation accuracy by refining pose sequences on a learned manifold with temporal regularization, yet the method still performs post-hoc refinement rather than enforcing explicit volumetric constraints.

With the development of deep learning, multi-view and volumetric methods have emerged to overcome the ambiguity of monocular inputs. Iskakov et al. [3] proposed a learnable triangulation framework that integrates 2D joint features from multiple cameras using a differentiable algebraic formulation, achieving significant accuracy improvements over single-view models. Garau et al. [22] and Arnab et al. [23] extended this concept by exploiting temporal consistency and/or jointly estimating camera parameters to refine the reconstructed 3D skeletons further. However, most of these methods still optimize losses on the image plane and depend on view-based projections rather than directly exploiting 3D volumetric information.

Recent frameworks such as EasyMocap [24] and ExPose [25] have sought to generate full-body 3D skeletons and parametric models, such as SMPL or SMPL-X, mainly from image observations or multi-view image constraints. EasyMocap fits a parametric body model to multiple calibrated views through reprojection optimization of 2D keypoints and model projections. However, it is limited by the accuracy of image-plane correspondences and calibration errors. Similarly, ExPose regresses expressive body, face, and hand parameters from a single RGB image in SMPL-X format via body-driven attention modules, rather than relying on multi-view consistency. Despite these advances, both approaches primarily rely on 2D projection losses or per-view consistency and thus cannot fully guarantee that the estimated joints lie strictly inside the physical body mesh, especially under occlusions or depth ambiguity. SMPL and SMPL-X fitting can also be applied directly to reconstructed 3D geometry, including point clouds or meshes, by optimizing body pose and shape parameters under surface proximity, articulation consistency, and learned body-prior constraints. Such approaches are highly relevant when a high-quality 3D reconstruction is available, because they can produce anatomically plausible full-body models as well as joint estimates. The proposed method differs from this line of work in that it does not estimate SMPL parameters or recover a parametric body surface. Instead, it extracts and refines sparse joint locations using virtual-plane projection, back-projection intersection, local vertex distributions, DBSCAN-based clustering, and geometric fitting. Therefore, our contribution should be understood as a training-free and geometry-driven skeleton extraction approach that is complementary to direct SMPL fitting, rather than as a general replacement for it.

In parallel, hybrid strategies that combine geometric priors, optimization, and learning-based inference have been increasingly explored in the field of human pose estimation. For example, integration of heatmap-based pose estimation with optimization-based refinement [18], geometry-aware feature refinement under occlusion [19], and temporal modeling with optimization-based correction [20] demonstrate the advantages of fusing domain knowledge with deep learning frameworks. These approaches highlight the effectiveness of constraining the solution space using structural priors while improving robustness through optimization.

In contrast to image-based and projection-limited pose estimation methods, the proposed approach directly utilizes the volumetric information of reconstructed 3D models obtained from multi-view RGB-D cameras. By combining PCA-based frontal alignment, virtual-plane projection, back-projection intersection, and density-based clustering with circle or sphere fitting, our method ensures that all estimated joints lie inside the reconstructed mesh volume. This strategy enforces explicit 3D consistency and reduces both mean joint error and its variance in the evaluated setting. The comparison with EasyMocap is presented as a limited reference comparison with an image-based multi-view SMPL pipeline, not as evidence of general superiority over all SMPL-based fitting approaches.

## 3. 3D Pose Estimation

This section describes our algorithm for 3D pose estimation. The algorithm consists of five steps: 3D point cloud reconstruction, multi-view projection of the point cloud, 2D pose estimation, 3D skeleton generation by joint intersection, and 3D skeleton refinement. The first step is the 3D reconstruction, which is introduced in Section 3.2. In the third step, we use OpenPose for 2D pose estimation, one of the most widely used techniques. We will explain the remaining three steps in this section.

### 3.1. Workflow

**Figure 1:**
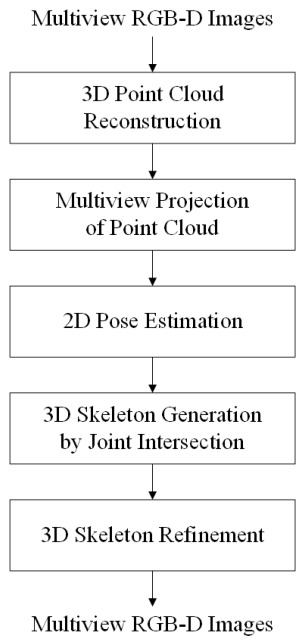
The proposed algorithm for 3D skeleton extraction.

After reconstructing the 3D point cloud (or mesh) from the multi-view RGB-D camera system, the four images are projected onto the four virtual planes from the 3D point cloud. Next, four 2D skeletons are estimated using OpenPose, and the intersection is calculated by back-projecting the joints of these skeletons into 3D space, including the 3D point cloud. The intersection corresponds to the 3D skeleton. Finally, the refinement process is applied to the 3D skeleton to improve its accuracy. The proposed algorithm is shown in Figure 1.

### 3.2. 3D Reconstruction

The 3D pose estimation proposed in this paper does not generate a skeleton directly from an object, but rather from a 3D point cloud reconstructed from the object using multi-view cameras. Therefore, we will explain the method to generate a 3D point cloud using multi-view RGB-D cameras.

**Figure 2:**
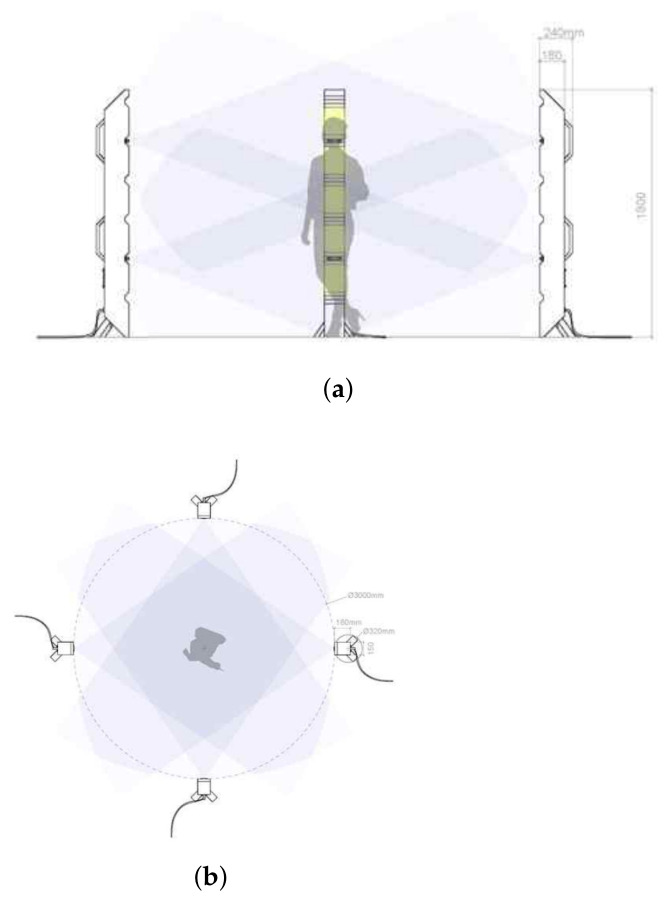
3D point cloud capturing system (**a**) vertical, (**b**) horizontal shooting angle and range.

To generate a photorealistic 3D model, we use an RGB-D camera with a 3D sensor. Our goal is to generate a volumetric 3D model; so, we install 8 RGB-D cameras at multiple viewpoints. Four stands with two cameras are positioned at the front, rear, left, and right sides of the scene. Figure 2 shows our camera system. Figure 2a is the capturing range of the side view, and Figure 2b is the capturing range of the top view. The 3D model capture method used in this paper has an average difference of 2.98 mm from the actual 3D model. It is also an exact method with a very low deviation of 3.39 mm [26].

We use feature points to calibrate multi-view point clouds, minimizing the distance between feature points extracted from different viewpoints. This is achieved by employing an optimization function to minimize distance errors between feature points. We use a Charuco board to find accurate feature points [27] rapidly and the gradient descent method for optimization [28].

The coordinates used in the optimization correspond to the internal corner coordinates of patterns on the Charuco board. The matrix for transforming feature points consists of six parameters for rotation and translation, corresponding to the *x*, *y*, and *z* axes. The rotation matrix and the translation matrix have the initial values before optimization. The transformation from one coordinate system to another is defined by Equation (1).

(1) $$X_{i}^{\prime }=R_{i\to ref}X_{i}+t_{i\to ref}$$

The loss (or error) function used in optimization is defined by the average sum of the squared Euclidean distance. The updating process is defined by Equation (2), where we find the global optimum with zero gradient.

(2) $$P_{n+1}=P_{n}-\alpha \frac{\partial }{\partial P_{n}}(\frac{1}{N}\sum_{j=0}^{N}∥X_{ref(j)}-X_{i}^{\prime }\left(j\right){∥}_{2}^{2})$$

The system outputs depth and RGB images from multiple RGB-D cameras, which are used for calibration. The RGB image is used to find the feature point using the Charuco board, and the depth image is used to acquire the 3D coordinates of the feature point. Then, a coordinate transformation parameter that minimizes the Euclidean square distance of the coordinates is calculated using gradient descent. When an extrinsic parameter is determined for each camera, point clouds generated in all camera coordinate systems can be unified into a standard coordinate system, referred to as a world coordinate system, and aligned in the same space [26].

### 3.3. Multi-View Projection

When estimating a 2D skeleton from a 3D model using OpenPose, the pose estimation of the front plane generally yields the most accurate results. Therefore, we find the front side of an object by analyzing the spatial distribution of the 3D model. We use the principal component analysis (PCA) to find the front side [29].

**Figure 3:**
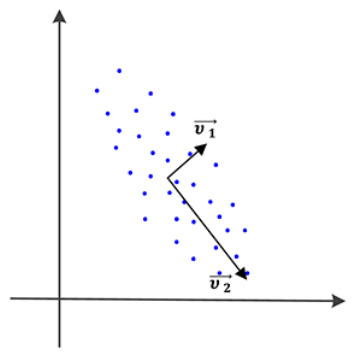
Example of PCA in 2D space.

Figure 3 shows two vectors $\vec{\nu_{1}}$ and $\vec{\nu_{2}}$ found using the PCA when data is distributed to an ellipse on a 2D plane. $\vec{\nu_{1}}$ and $\vec{\nu_{2}}$ mostly represent the distribution characteristics of the data. If you find the direction and size of these vectors, you can effectively analyze the shape of the data distribution [29].

**Figure 4:**
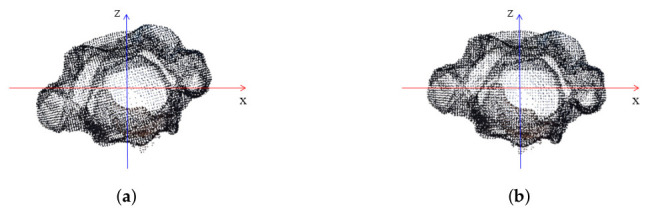
Object rotation (**a**) before and (**b**) after using PCA.

To estimate the front direction of the 3D model, PCA is applied to the projected points on the xz-plane, excluding the vertical (*y*) direction. Among the principal components, the eigenvector corresponding to the smallest eigenvalue is selected as the front direction, as illustrated in Figure 4. The 3D model is then rotated so that this direction aligns with the z-axis, facilitating the definition of virtual projection planes.

**Figure 5:**
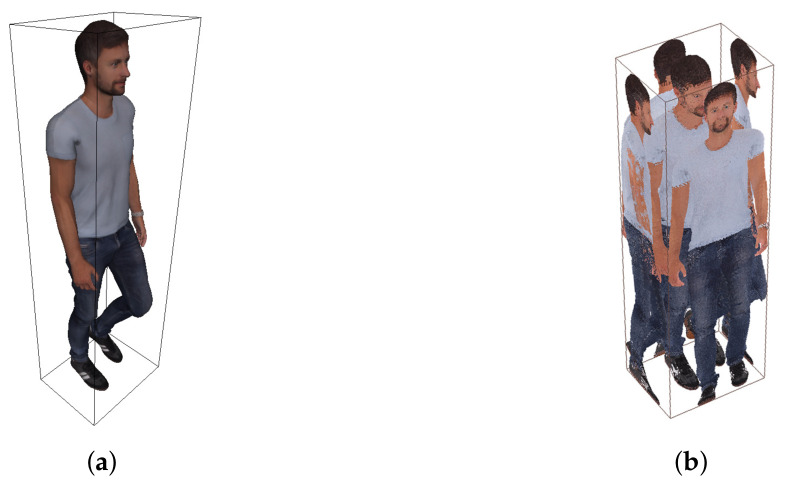
Projection method from 3D point cloud to 2D images (**a**) definition of the AABB, (**b**) projection result on the four sides.

After alignment, an axis-aligned bounding box (AABB) is constructed to define the projection space. The 3D model is projected onto virtual planes using a model-view projection matrix, which transforms world coordinates into the corresponding 2D plane coordinates. The projection process onto multiple sides of the AABB is illustrated in Figure 5. The resolution of the virtual plane projection is adaptively determined based on the size of the AABB of the reconstructed 3D model. Instead of using a fixed image resolution, the spatial extent of the AABB is mapped to the pixel grid, allowing the projected image to preserve the geometric scale and point density. The projection is performed using a model-view projection matrix after PCA-based alignment, and the coordinates are normalized and quantized into pixel coordinates. The RGB values are directly mapped from the point cloud without additional rendering or shading processes.

Figure 5b is the result of transforming into a pixel coordinate system and projecting it into a 2D image. The four 3D images are projected on the four sides (front, rear, right, left side) in Figure 5b. To transform the projected object into a pixel coordinate system, the dynamic range is changed, and quantization is performed to an integer type.

### 3.4. 3D Skeleton Generation

**Figure 6:**
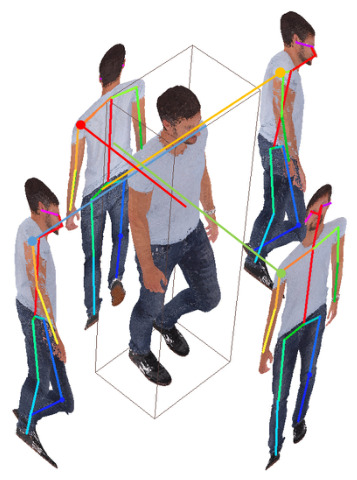
Example of point cloud’s left shoulder 3D joint extraction.

After generating four projected images, the 2D pose is estimated using OpenPose [30]. Figure 6 shows the 2D skeleton and the projected image on the four planes. There is no limitation for a 2D pose estimation algorithm. The improvement in 2D pose performance may enhance the proposed algorithm’s performance.

The 3D model is projected onto four 2D images, and the 2D skeleton on these images is back-projected into the 3D space. Through this process, we can obtain the 3D joint of the 3D model. The joints on the four 2D images are vertically projected from the plane with an inner direction. The virtual projection lines of the four joints included in the four 2D skeletons intersect in 3D space. The intersection can be regarded as a 3D joint of the 3D model. This process is depicted in Figure 6.

**Figure 7:**
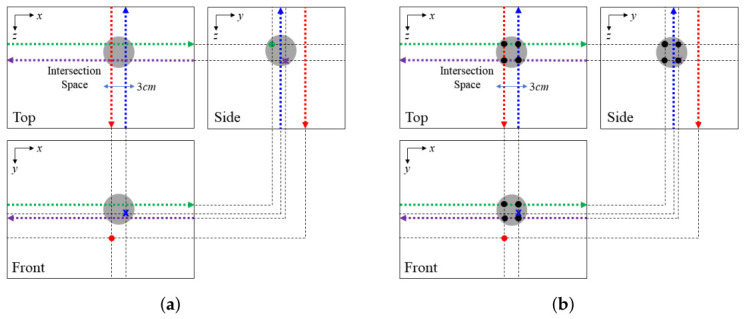
Error joint removement and 3D joint generation (**a**) intersection definition by joint projection, (**b**) 3D coordinate definition for generating a 3D joint.

In general, 2D pose estimation has an error, and due to this error, a projection line may be far from the intersection space. As illustrated in Figure 7a, when the red projection line on the rear side is examined in the front and side views, it falls outside the intersection space. The diameter of the intersection space was determined considering the physical size of human joints. In general, the spatial extent of major human joints such as shoulders, elbows, and knees is typically within approximately 5 cm. To impose a stricter constraint and reduce the effect of 2D pose estimation noise, we selected a smaller diameter of 3 cm. This conservative threshold helps reject outliers while maintaining stable and accurate 3D joint estimation. That is, after defining a 3D virtual sphere, if the virtual projection line does not pass through this space, the source joint of this virtual projection line is excluded from the calculation to incorporate the 3D joint. The average coordinates are calculated after defining 4 points in 3 views for the 3D joint using candidate coordinates that are not removed. The coordinates of (*x*, *z*) are determined in the top view, and the *y* coordinates are determined in the side view. The calculated (*x*, *y*, *z*) coordinates must match the (*x*, *y*) coordinates in the front view. This process is shown in Figure 7b.

### 3.5. 3D Skeleton Refinement

**Figure 8:**
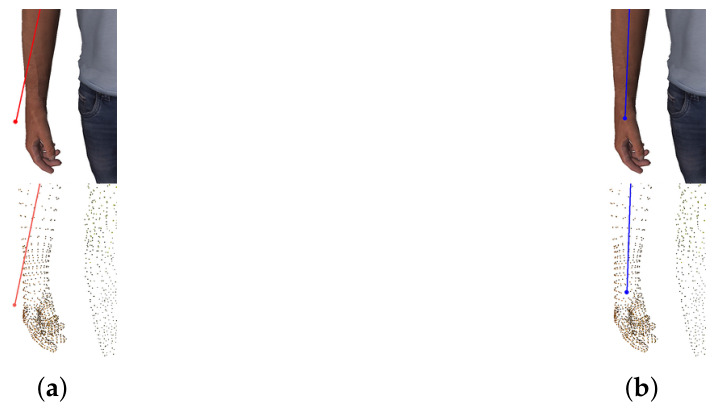
Point cloud and incorrectly extracted joint (**a**) an outlier of a joint, (**b**) a refined joint after refinement process.

A 2D pose estimation algorithm can generate incorrect joints because of various reasons, which leads to errors in the 3D joints calculated by our algorithm. This requires a refinement process. Figure 8a shows an example of the incorrect estimation of a joint in a 3D space. We can decide which joint has the incorrect position using a reconstructed volumetric 3D model. We propose a refinement algorithm that moves an outlier of a joint shown in Figure 8a to the appropriate 3D position of Figure 8b using the 3D skeleton refinement step in Figure 1.

**Figure 9:**
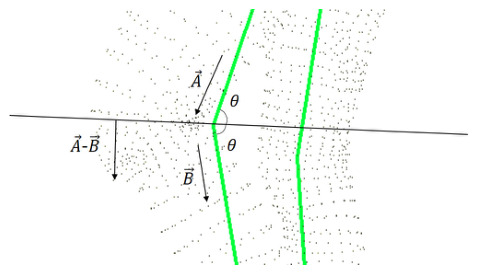
Definition of the plane through the bisector of the bones connected to the joint.

We define a virtual plane using a target joint and the direction of a bone relative to the joint, and combine the plane with points within a 3 cm distance. The distance for the threshold is selected by experiment. We cluster a valid set from points for the joint. The plane is defined as the bisector of two bones connected to the joint, shown in Figure 9.

**Figure 10:**
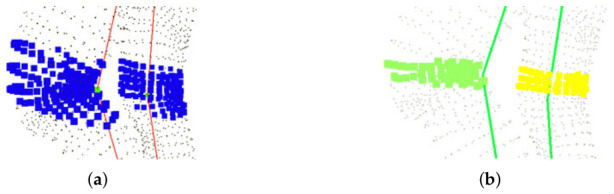
Point cloud for objects and (**a**) point set before clustering, (**b**) point set after clustering.

We use the density-based spatial clustering of applications with noise (DBSCAN) for clustering point clouds based on data density. DBSCAN has high accuracy for data with a random distribution. Since it can also distinguish noise from point clouds, it has an additional effect, such as noise reduction, when applied to the captured 3D point cloud with noise, unlike the graphic model [31]. Figure 10 shows the definition of the point set before and after clustering. After considering the direction of bones between joints, the point set is clustered around the target joint. Figure 10a shows the candidate model for clustering, and Figure 10b shows two point sets for joints of the left and right knee.

We use the center of each cluster as the refined position of a joint. To find the center of a cluster, circle fitting is applied to the cluster. If the center coordinate of a circle is $\left(x_{c},y_{c},z_{c}\right)$, the circle with the least error is defined by Equation (3) for *n* points $\left(x_{i},y_{i},z_{i}\right)$ in the cluster.

(3) $${\left(x_{i}-x_{c}\right)}^{2}+{\left(y_{i}-y_{c}\right)}^{2}+{\left(z_{i}-z_{c}\right)}^{2}=r^{2}$$

Using $W\left(w_{o},w_{1},w_{2},w_{3}\right)=\left(2x_{c},2y_{c},2z_{c},\left(r^{2}-x_{c}^{2}-y_{c}^{2}-z_{c}^{2}\right)\right)$, Equation (3) is rewritten as Equation (4).

(4) $$\left(x_{i},y_{i},z_{i},1\right)*W=x_{i}^{2}+y_{i}^{2}+z_{i}^{2}$$

For *n* points, Equation (4) is expressed as Equations (5)–(7).

(5) $$\begin{array}{c} AW \end{array}=B$$

(6) $$A=\left[\begin{array}{cccc} x_{1} & y_{1} & z_{1} & 1\\ x_{2} & y_{2} & z_{2} & 1\\ & \cdots & & \\ x_{n} & y_{n} & z_{n} & 1 \end{array}\right]$$

(7) $$B=\left[\begin{array}{c} x_{1}^{2}+y_{1}^{2}+z_{1}^{2}\\ x_{2}^{2}+y_{2}^{2}+z_{2}^{2}\\ \cdots \\ x_{n}^{2}+y_{n}^{2}+z_{n}^{2} \end{array}\right]$$

Among the possible *W* for Equation (5), $\hat{W}$, which makes the smallest error, can be obtained through Equation (8).

(8) $$\hat{W}={\left(A^{T}A\right)}^{-1}A^{T}B$$

**Figure 11:**
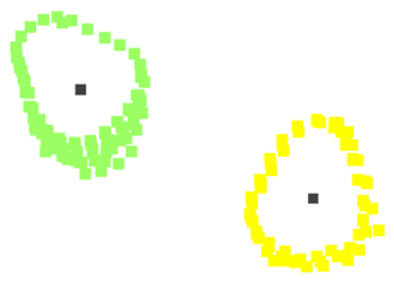
Center of cluster obtained using circle fitting.

Figure 11 is the center of the cluster calculated through Equation (8).

## 4. Experimental Result

### 4.1. Environment

We employed eight Microsoft Azure Kinect RGB-D sensors for volumetric capture. The cameras were arranged on four vertical stands positioned at the front, rear, left, and right sides of the capture volume. Each stand mounted two cameras at different heights, with lower cameras placed at 0.7 m and upper cameras at 1.5 m from the ground. The cameras were positioned at distances of approximately 1 m to 2 m from the target object. In addition, distances below 1 m can introduce depth errors when using RGB-D sensors, which may degrade the reconstruction quality. Therefore, the capture setup was designed to maintain a distance of at least 1 m to ensure stable and accurate depth measurements The calibration accuracy achieved a mean registration error of 2.98 mm with a standard deviation of 3.39 mm [26]. The experiments were conducted in the volumetric capture studio at Kwangwoon University. The processing system utilized a dual-workstation configuration. The first workstation handled point cloud generation and registration, while the second workstation was dedicated to hologram generation. Each workstation was equipped with two NVIDIA GeForce RTX 2080 GPUs, enabling real-time generation of 2K FHD color holograms at 30 fps.

**Figure 12:**
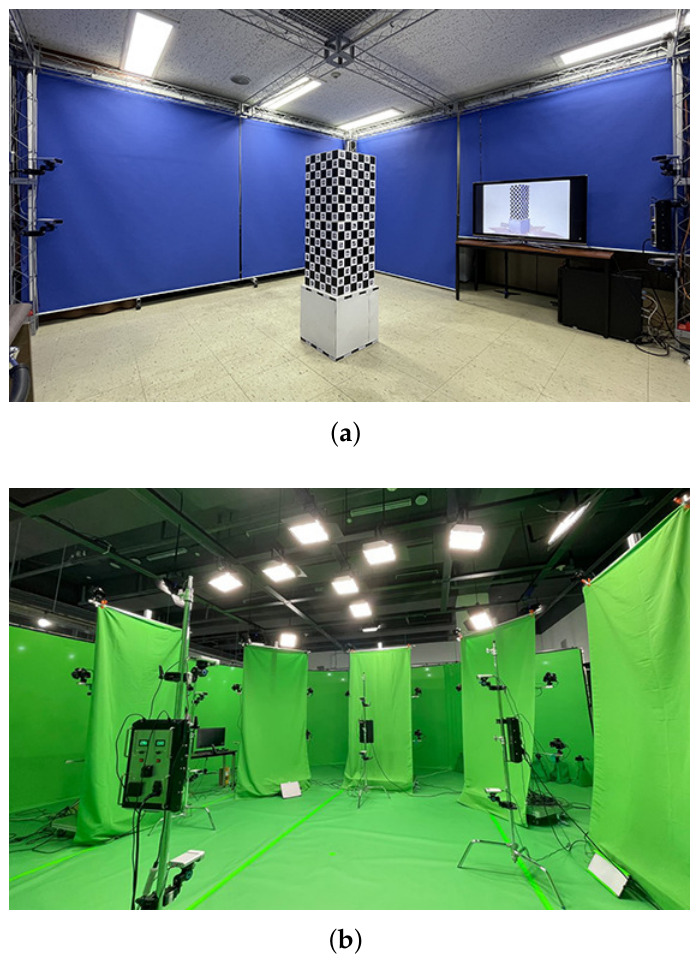
Camera system environment used (**a**) RGB-D based capturing system, (**b**) volumetric capturing studio.

The capturing environment is shown in Figure 12. We used a capturing system using eight RGB-D cameras. The arrangement of the system is shown in Figure 12a. Four cameras were installed at a height of about 0.7 m from the ground, and the others were installed at a height of about 1.5 m from the ground. The cameras were positioned at a distance of 0.1 m to 2 m from the center. The next capturing environment is a volumetric capturing studio, which features 52 4K and 8K cameras, as shown in Figure 12b for comparison with optical sensor-based motion capturing. This system reconstructs a 3D volumetric model using photogrammetric methodology.

### 4.2. Result of 3D Reconstruction

Each camera outputs RGB and depth images at a speed of 30 fps. The point clouds from eight viewpoints, each with its own camera coordinate system, can be generated using two types of images and intrinsic parameters. After eight sets of point clouds are generated, these are registered to an integrated point cloud through a camera calibration process. The average calibration error of the point cloud was measured to be approximately 2.98 mm.

**Figure 13:**
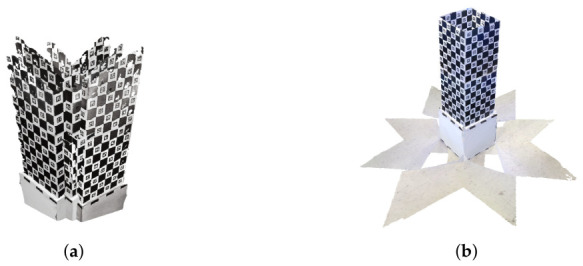
Point cloud before and after integration (**a**) point cloud output from each camera, (**b**) point cloud integrated through coordinate transformation parameters.

Figure 13 shows the result before and after calibration for the Charco box of Figure 12a. Figure 13a shows point clouds by each camera before calibration, and Figure 13b shows the integrated point cloud after calibration.

### 4.3. 3D Skeleton Extraction Result

The experiment on 3D pose estimation was performed using 3D models with ground truth [32]. The error for joints was calculated through MPJPE (Mean per Joint Position Error). MPJPE calculates the average error between the ground truth joints and the estimated joints using the proposed method, which is defined as in Equation (9) [33].

(9) $$E_{MPJPE}\left(p_{o},\hat{p_{o}}\right)=\frac{1}{N}\sum_{i=1}^{N-1}{∥p_{o}^{i}-\hat{p_{o}^{i}}∥}_{2}$$

In Equation (9), $p_{o}$ is a joint coordinate of the ground truth, and $\hat{p_{o}}$ is a joint coordinate of the estimated skeleton. *N* is the number of joints, and we use 15 joints. We verified the performance of our algorithm with the MPJPE. The definition of joint positions may vary according to the pose estimation algorithm. Therefore, we calculated the standard deviation (SD) of the MPJPE to verify how stably the 3D pose can be estimated through the proposed algorithm.

**Figure 14:**
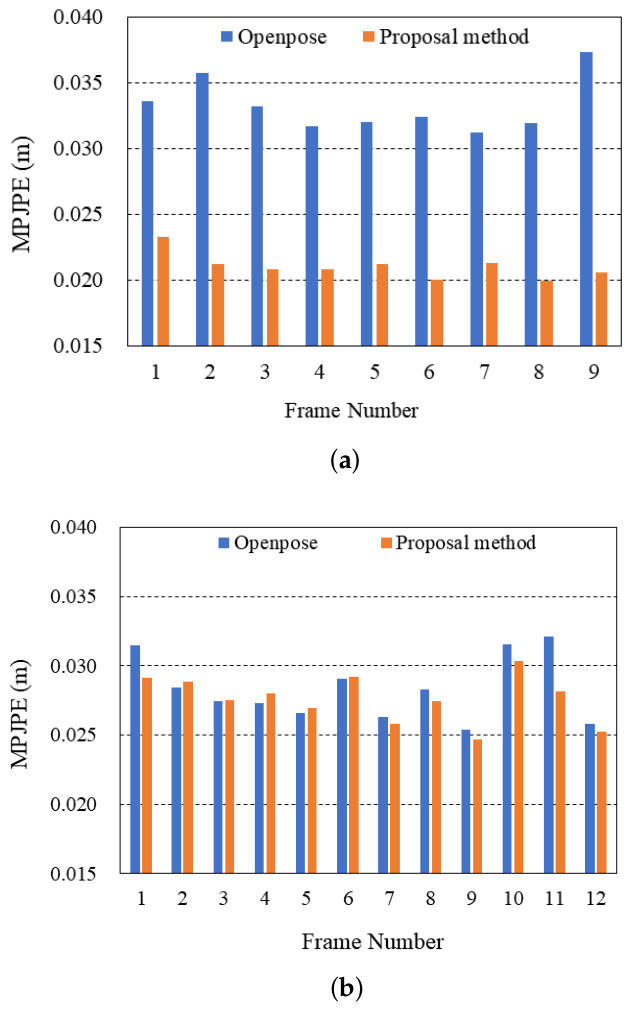
PMPJPE results for each frame before and after correction (**a**) Eric, (**b**) Sophia.

Figure 14 shows the quantitative evaluation results obtained by measuring the MPJPE using the ground-truth joints of the rigged 3D models. Figure 14 also presents frame-by-frame MPJPE values to illustrate how the estimation error varies over time. The displayed frames correspond to consecutive samples selected from the test sequence, and the values represent per-frame joint errors rather than aggregated statistics. Therefore, the results in this figure reflect temporal variations in the estimation performance. The results in Figure 14 are obtained from consecutive frames in the evaluation sequence, and the displayed frames are selected as representative samples to illustrate the effect of the proposed refinement process. Figure 14a corresponds to Eric and Figure 14b to Sophia. For the Eric model, the proposed method achieved an average MPJPE of 33.2 mm before refinement (OpenPose baseline) and 1.93 mm after refinement, resulting in an improvement of approximately 31.28 mm in mean error and a 7.8% reduction in standard deviation. For the Sophia model, the mean MPJPE decreased from 21.0 mm to 0.92 mm, indicating an improvement of 0.18 mm and a 5.9% reduction in standard deviation. These results indicate a consistent trend of both lower mean error and lower variance after applying the proposed virtual-projection and refinement pipeline. Although the absolute MPJPE difference is partly influenced by how each algorithm defines joint positions, the relative trend demonstrates that the proposed method produces more stable joint localization across frames.

We quantitatively evaluated the stability and accuracy of the proposed method by calculating both the mean and standard deviation (SD) of the MPJPE values, since the joint definition criteria may differ across algorithms. Table 1 summarizes the detailed results for the Eric (69 frames) and Sophia (617 frames) models, where OpenPose was used to obtain the initial skeletons from the projected 2D planes. In contrast to Figure 14, Table 1 summarizes the overall performance by reporting the temporal mean and standard deviation of MPJPE across all frames and joints. The mean value represents the average joint error over the entire sequence, while the SD indicates the stability of the estimation over time. Since Table 1 provides aggregated statistics and Figure 14 shows per-frame variations, the numerical values are not directly comparable. For the Eric model, the mean MPJPE decreased from 33.21 mm (OpenPose) to 1.93 mm after refinement, and the SD decreased from 28.33 mm to 2.22 mm, corresponding to reductions of 5.81% and 7.83%, respectively. For the Sophia model, the mean error improved from 21.02 mm to 0.92 mm, and the SD from 27.63 mm to 1.63 mm, which represent reductions of 4.36% and 5.89%, respectively. These numerical results indicate a clear trend that the proposed volumetric refinement pipeline consistently reduces both the average joint error and its variability. In particular, the low SD values demonstrate that the proposed method achieves stable joint localization across frames, while maintaining high accuracy even under different body shapes or capture conditions. The trend confirms that enforcing volumetric consistency through virtual-plane projection and DBSCAN-based refinement effectively improves both precision and robustness of 3D joint estimation.

**Figure 15:**
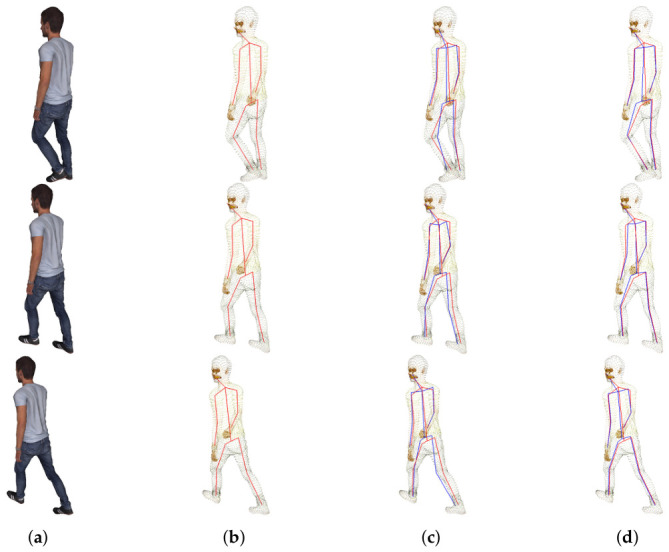
(**a**) mesh, (**b**) point cloud and ground truth skeleton, (**c**) point cloud, ground truth skeleton, skeleton before correction, (**d**) point cloud, ground truth skeleton, after correction skeleton of Eric.

**Figure 16:**
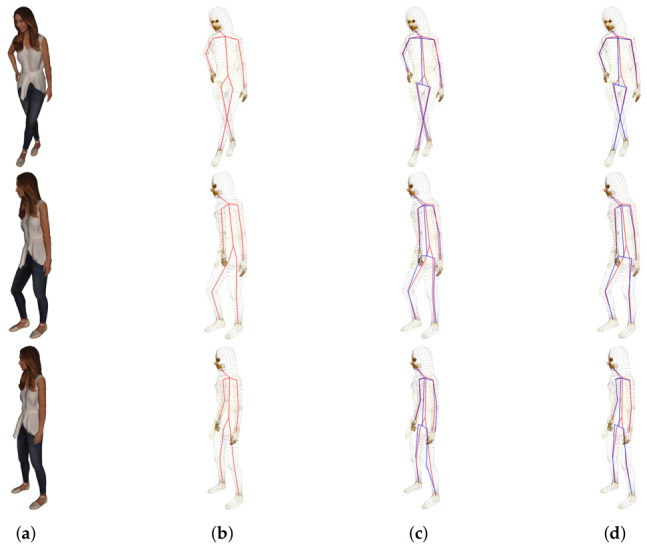
(**a**) mesh, (**b**) point cloud and ground truth skeleton, (**c**) point cloud, ground truth skeleton, skeleton before correction, (**d**) point cloud, ground truth skeleton, after correction skeleton of Sophia.

Figure 15 and Figure 16 visually display the pose estimation results for the three frames of Eric and Sophia objects on a 3D model. In both figures, (a) is a 3D model, and the red color in (b) is a skeleton corresponding to the ground truth. (c) shows the skeleton we obtained in blue. Examining the results for the first frame in Figure 15c, it is evident that the joint for the left leg is not accurate. It is confirmed that the joint of the left leg deviates to the external area of the object. Here, the result after performing the correction algorithm is (d). Examining the results for the first frame in Figure 15d, it is evident that the error for the left leg has nearly disappeared. In the second and third frames of Figure 15c, it can be seen that some errors occurred in the joint of the right ankle. It is observed that a part of the skeleton deviates from the outside of the 3D object due to an error in the joint of the right ankle. After applying the refinement algorithm, it can be seen that all these errors are improved in the results of Figure 15d.

The results for Sophia in Figure 16 are slightly better than those in Figure 15. In the first and second frames, there was a relatively small error in the joints of both ankles, and in the third frame, an error occurred in the left knee. However, the error is relatively small for all frames.

We also conducted quantitative experiments comparing our method with existing approaches. Although recent studies directly addressing volumetric data and corresponding 3D Body25 joint datasets are limited, we used EasyMocap, a representative method that effectively fits SMPL models from multi-view images, as a baseline for quantitative comparison. The comparison was performed using the Body25 regressor adopted by EasyMocap, and the results are shown in Table 2. For EasyMocap, MPJPE values exceeded 100 mm for both the Sophia and Eric models. In contrast, our method achieved 23.23 mm and 33.56 mm, respectively, yielding a stable average MPJPE of 28.40 mm. This difference can be partly attributed to the different use of geometric information: EasyMocap primarily relies on multi-view image observations and model projection constraints, whereas the proposed method directly uses the spatial distribution of vertices in the reconstructed volumetric model. Therefore, the results in Table 2 should be interpreted as a limited reference comparison showing the effectiveness of volumetric geometric constraints in the evaluated setting. They should not be interpreted as evidence of a general advantage over SMPL-based approaches, especially methods that directly fit SMPL or SMPL-X to the same reconstructed 3D point cloud or mesh. A direct comparison with such methods would require unified joint definitions, coordinate systems, fitting objectives, and evaluation protocols, and is left for future work.

To further evaluate the generalizability of the proposed method, we conducted additional experiments using three public datasets: EgoHumans, MPI-INF-3DHP, and Panoptic. Table 3 summarizes the quantitative evaluation results, including mean error and standard deviation. The proposed method achieved mean errors of 33.403 mm, 69.738 mm, and 30.736 mm for EgoHumans [34], MPI-INF-3DHP [35], and Panoptic [36], respectively, with relatively low standard deviations of 8.226 mm, 18.533 mm, and 3.803 mm. These results demonstrate that the proposed algorithm provides stable and accurate 3D joint estimation across datasets with different capture environments and motion characteristics. In particular, the low standard deviation observed in the EgoHumans and Panoptic datasets indicates that the proposed volumetric intersection and refinement strategy consistently improves joint localization accuracy. Overall, these experimental results confirm that the proposed method maintains robust performance and strong generalizability across diverse datasets.

### 4.4. Joint Error Refinement

**Figure 17:**
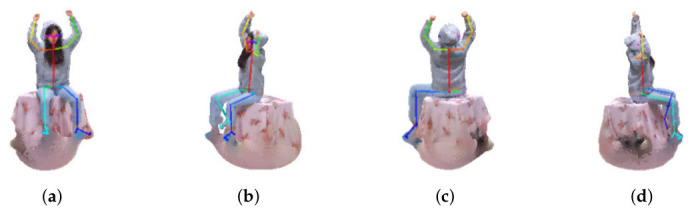
Extraction of the 2D skeleton of the projected image (**a**) front, (**b**) right, (**c**) rear, (**d**) left.

**Figure 18:**
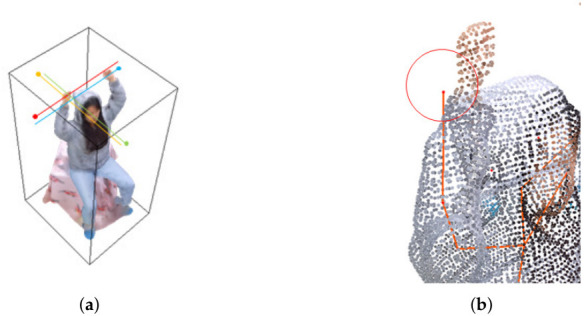
The extracted joint with error (**a**) joint intersection, (**b**) incorrect joint.

We show a pose estimation result of the 3D point cloud captured by our system. Since the captured 3D point cloud lacks a ground truth of the 3D skeleton, it is challenging to evaluate its numerical quality. Therefore, we discuss the subjective evaluation by visual observation. Figure 17 shows the 2D skeleton results estimated using four 2D images projected from the 3D point cloud. In Figure 18, we can visually find some joint error. We deal with the joint of the right wrist.

The estimated 3D joint is shown in Figure 18. After calculating the intersection using four joints of Figure 18 in 3D space as shown in Figure 18a, the resultant joint can have an error as shown in Figure 18b. We can identify that the joint of the right wrist is located outside the 3D point cloud.

**Figure 19:**
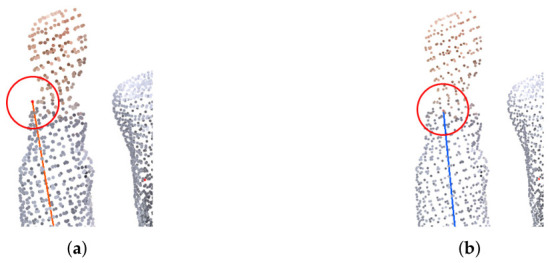
Joints that are located outside the object (**a**) before correction, (**b**) moved inside the object after correction.

Next, by analyzing the distribution of the 3D point cloud and adjusting the position of the joint to the center of the 3D point cloud, the correct joint can be obtained. Figure 19a is the result before correction, and Figure 19b is the result after correction. Since there is no ground truth for the acquired object, it is difficult to confirm how quantitatively the correct result is in Figure 19b compared to Figure 19a. We can predict the superiority of the results by relying on the subjective visual evaluation and the results of previous quantitative experiments.

To analyze the effect of depth blurring and occlusion, we conducted an additional experiment by varying the number of viewpoints. Table 4 shows the MPJPE results for the left shoulder joint as the number of viewpoints increases from one to four. When using a single viewpoint, the MPJPE was 15.59 mm. As the number of viewpoints increased to two, three, and four, the MPJPE decreased to 12.78 mm, 10.47 mm, and 10.46 mm, respectively. This corresponds to an overall error reduction of approximately 32.9 % from one to four viewpoints. These results indicate that increasing the number of viewpoints reduces depth ambiguity and occlusion effects. With multiple viewpoints, projection rays intersect more densely within the valid body volume, allowing for more accurate joint localization. Consequently, the proposed volumetric constraint framework improves geometric consistency and alleviates depth blurring and occlusion problems.

### 4.5. Comparison with Motion Capture

To evaluate the accuracy of the reconstructed 3D models, we compared the joint positions obtained from a motion capture system with those estimated by the proposed method. Since ground-truth joint annotations are not available for the reconstructed models, direct computation of MPJPE with respect to ground truth is not feasible. Instead, we analyzed the temporal consistency between the two measurements. Due to differences in joint definitions—where motion capture joints correspond to sensor locations on the body surface, while the proposed method estimates joints within the body volume—the absolute joint positions are not directly comparable. Therefore, we evaluated the temporal stability of the positional differences between the two methods using the temporal standard deviation (TSD). We used the Perception Neuron 32 V2 motion capture system [37] and measured 15 joints [38] over several hundred frames. The results, shown in Table 5, indicate that the average TSD values are 3.111 mm for Sol and 3.441 mm for Sun-Jong, demonstrating high consistency between the two methods.

**Figure 20:**
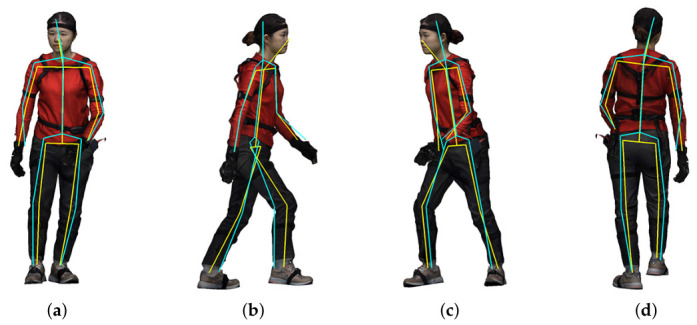
Estimated 3D joint and bones using a motion capture sensor and the proposed method (**a**) front, (**b**) left, (**c**) right, and (**d**) rear view.

Figure 20 shows four viewpoints of a reconstructed 3D volumetric model captured using the system illustrated in Figure 12b. The joint positions obtained from the motion capture system and the proposed method are visualized on the point cloud, where the green skeleton represents the motion capture results and the yellow skeleton represents the proposed method. Since motion capture joints correspond to sensor locations attached to the body surface, they may appear outside the body depending on the viewpoint. In contrast, the proposed method estimates joint positions based on volumetric geometry, resulting in joints located within the body. This difference reflects the distinct definitions of joint positions rather than an estimation error.

As observed in Figure 20, the definitions of the joint locations of the two methods differ. Therefore, the resultant 3D skeletons have different spatial features. This difference is a natural phenomenon. As previously explained, the spatial difference should exhibit temporal consistency, and this condition was satisfied by the measured TSD results of approximately 3 mm, as shown in Table 5. As shown in Figure 20, the motion capture sensors are attached to the body using visible black straps, and thus their positions are located on or slightly outside the surface of the human body. In contrast, the joints estimated by vision-based methods, including the proposed approach, are defined within the body volume based on geometric or anatomical assumptions. Therefore, the apparent discrepancy between the two results originates from the difference in joint definitions rather than estimation error.

### 4.6. Comparison with Other Research

To provide additional context for the proposed method, we compared it with recent 3D pose estimation algorithms published in the past three years, including ActionPose [13], RePOSE [11], and PersPose [12]. As shown in Table 6, ActionPose and RePOSE achieved MPJPE values of 15.5 mm using supervised learning with ground-truth 2D pose sequences, while PersPose reported 72.1 mm using monocular RGB images. The proposed method achieved an MPJPE of 69.7 mm without any additional training, outperforming PersPose by 2.4 mm. Unlike learning-based approaches, the proposed method uses volumetric constraints derived from reconstructed 3D human models. By restricting joint candidates within the valid body volume and refining them through clustering, the proposed method improves geometric consistency and reduces outliers. These results provide a contextual reference showing that the proposed volumetric constraint framework can produce stable results while maintaining a training-free, geometry-driven approach. Because these methods use different input modalities, supervision levels, and evaluation protocols, the comparison should be regarded as a contextual reference rather than a direct apples-to-apples benchmark.

The proposed method focuses on improving geometric consistency and accuracy through volumetric constraints, rather than real-time performance. Due to the multi-stage pipeline, including projection, intersection, and refinement processes, the current implementation is not designed for real-time applications. Instead, it is intended for high-precision offline analysis. Future work will investigate optimization strategies to reduce computational complexity and enable real-time or near real-time processing.

## 5. Discussion & Remarks

### 5.1. Discussions

The observed stability and geometric consistency of the proposed method can be attributed to the direct utilization of volumetric information derived from reconstructed 3D models. Unlike image-based approaches, our method leverages the spatial distribution of vertices to constrain joint positions within geometrically valid regions. Unlike parametric model-fitting approaches, it does not optimize predefined body shape and pose parameters, but directly refines sparse joint locations from the reconstructed 3D geometry. In particular, volumetric data provide rich information such as the spatial distribution of vertices, which cannot be obtained solely from conventional multi-view images. Based on this, the PCA-based frontal alignment and virtual-plane projection minimize occlusion issues and clearly separate depth ordering, thereby constraining joint candidates within a physically consistent region. In the subsequent back-projection process, multi-view intersections are integrated, and outliers are effectively eliminated by combining bone-direction analysis with density-based clustering. Some image-plane-based methods may have limited ability to correct outliers located outside the reconstructed body volume, because their optimization is mainly performed through projection or reprojection constraints. In contrast, the proposed method explicitly uses local volumetric geometry to refine such joints inside the reconstructed body region. Therefore, the proposed use of volumetric data contributes to reductions in both mean error and variability in the evaluated experiments.

### 5.2. Remarks

What should be emphasized in this study is that 3D consistency should not merely be achieved through inter-view alignment but should instead be directly ensured within the volumetric domain, which contains rich spatial information. The proposed virtual-plane projection serves as an interpretable bridge between 3D geometry and 2D joint detection, automatically eliminating unnecessary joint candidates and producing structurally reliable results. Furthermore, the proposed framework can maintain its overall architecture even when employing different 2D pose detectors (e.g., R-CNN [39]) or alternative joint definition schemes (e.g., COCO17 [40]), demonstrating strong scalability to various sensor environments and motion capture systems in future applications.

## 6. Conclusions

In this paper, we proposed a training-free, geometry-driven 3D human pose estimation algorithm that directly utilizes the volumetric information of reconstructed 3D models obtained from a multi-view RGB-D camera system. Our method differs from parametric model-fitting approaches in that it does not estimate predefined body-model parameters, but instead extracts and refines sparse joint positions from reconstructed volumetric data. This highlights a complementary direction to SMPL-based fitting in 3D pose estimation research. The proposed algorithm consists of five main stages: 3D model reconstruction, virtual-plane projection, 2D pose estimation, 3D joint computation through back-projection, and density-based refinement. These processes effectively alleviate the depth ambiguity and occlusion problems commonly found in image-plane-based methods, enabling the reconstruction of highly accurate 3D poses while maintaining the physical consistency of joints. Quantitative experiments demonstrated that the proposed method reduced both the mean error and standard deviation by up to 7.83% when evaluated with rigged 3D models, resulting in more stable and accurate estimations compared to conventional OpenPose-based approaches. In addition, the comparison with a multi-view SMPL-based image pipeline provides a limited reference showing that direct use of reconstructed volumetric geometry can be effective in the evaluated setting. However, this result should not be interpreted as a general superiority claim over SMPL-based approaches, especially those that directly fit parametric body models to the same 3D point cloud or mesh. The subjective visual evaluation also confirmed that the refinement algorithm successfully positioned the estimated joints inside the object surface. In conclusion, this study presents a new direction for pose estimation using volumetric data, demonstrating robust 3D joint estimation even in complex scenes or occluded environments where conventional methods often fail. Future work will focus on conducting a direct benchmark against SMPL and SMPL-X fitting methods applied to the same reconstructed 3D point clouds or meshes, using unified joint definitions, coordinate systems, and evaluation metrics. We will also investigate integration with parametric body models to broaden the applicability of the proposed framework to animation production, motion analysis, and metahuman rigging.

## Figures and Tables

**Table 1:** Frame-by-frame MPJPE results before and after correction, standard deviation and average value.

Model	Method	Value (mm)		Enhanced Ratio (%)	
		Mean	SD	Mean	SD
Eric	Openpose	33.215	28.325	100.00%	100.00%
	Proposed	1.929	2.217	5.81%	7.83%
Sophia	Openpose	21.015	27.626	100.00%	100.00%
	Proposed	0.916	1.627	4.36%	5.89%

**Table 2:** MPJPE measurement results of the Eric and Sophia models comparing EasyMocap (multi-view-based) with our proposed method.

Method	MPJPE (mm) ↓		Avg. MPJPE (mm) ↓
	Sophia	Eric	
EasyMocap [24]	108.13	117.61	112.87
Ours	23.23	33.56	28.40

**Table 3:** Quantitative evaluation results on three datasets.

Dataset	Mean (mm)	SD (mm)
EgoHumans [34]	33.403	8.226
MPI-INF-3DHP [35]	69.730	18.533
Panoptic [36]	30.736	3.803

**Table 4:** MPJPE of the left shoulder according to the number of viewpoints.

Number of View Point	MPJPE (mm)
1	15.59
2	12.78
3	10.47
4	10.46

**Table 5:** Accuracy comparison by the temporal standard deviation of the error distance (unit: mm) between the proposed method and the motion capture sensor.

Index	Joint	Sol	Sunjong
0	Head	2.980	3.435
1	Neck	2.345	3.361
2	Right Shoulder	4.330	5.098
3	Right Elbow	3.976	0.644
4	Right Wrist	3.318	4.694
5	Left Shoulder	2.380	5.185
6	Left Elbow	2.825	1.125
7	Left Wrist	1.932	6.392
8	Pelvis	3.007	5.196
9	Right Hip	4.751	1.310
10	Right Knee	1.999	2.565
11	Right Ankle	2.315	1.880
12	Left Hip	4.625	3.649
13	Left Knee	3.436	2.889
14	Left Ankle	2.441	4.195
Average		3.111	3.441

**Table 6:** Comparison with recent state-of-the-art 3D pose estimation methods on MPI-INF-3DHP dataset.

Method	Year	Input Type	MPJPE (mm)
ActionPose [13]	2024	2D Pose Sequence (GT)	15.5
RePOSE [11]	2024	2D Pose Sequence (GT)	15.5
PersPose [12]	2025	RGB Image	72.1
Ours	2026	2D pose Sequence (GT)	0.18
		RGB Image	69.7

## Data Availability

Data are contained within this article.
